# Chloroplastic biosynthesis of melatonin and its involvement in protection of plants from salt stress

**DOI:** 10.1038/srep41236

**Published:** 2017-02-01

**Authors:** Xiaodong Zheng, Dun X. Tan, Andrew C. Allan, Bixiao Zuo, Yu Zhao, Russel J. Reiter, Lin Wang, Zhi Wang, Yan Guo, Jingzhe Zhou, Dongqian Shan, Qingtian Li, Zhenhai Han, Jin Kong

**Affiliations:** 1 College of Horticulture, China Agricultural University, Beijing, China; 2 Department of Cellular & Structural Biology, The UT Health Science Center, San Antonio, TX, USA; 3 Plant & Food Research, Private Bag 92169, Auckland, New Zealand; 4 College of Biological Sciences, China Agricultural University, Beijing, China

## Abstract

Within the chloroplasts reactive oxygen species (ROS) are generated during photosynthesis and stressful conditions. Excessive ROS damages chloroplasts and reduces photosynthesis if not properly detoxified. In this current study, we document that chloroplasts produce melatonin, a recently-discovered plant antioxidant molecule. When *N*-acetylserotonin, a substrate for melatonin synthesis, was fed to purified chloroplasts, they produced melatonin in a dose-response manner. To further confirm this function of chloroplasts, the terminal enzyme for melatonin synthesis, N-acetylserotonin-O-methyltransferase (ASMT), was cloned from apple rootstock, *Malus zumi.* The *in vivo* fluorescence observations and Western blots confirmed MzASMT9 was localized in the chloroplasts. A study of enzyme kinetics revealed that the *K*_m_ and *V*_max_ of the purified recombinant MzASMT9 protein for melatonin synthesis were 500 μM and 12 pmol/min·mg protein, respectively. *Arabidopsis* ectopically-expressing *MzASMT9* possessed improved melatonin level. Importantly, the *MzASMT9* gene was found to be upregulated by high light intensity and salt stress. Increased melatonin due to the highly-expressed *MzASMT9* resulted in *Arabidopsis* lines with enhanced salt tolerance than wild type plants, as indicated by reduced ROS, lowered lipid peroxidation and enhanced photosynthesis. These findings have agricultural applications for the genetic enhancement of melatonin-enriched plants for increasing crop production under a variety of unfavorable environmental conditions.

Chloroplasts are critically important plant cellular organelles with the major function of performing photosynthesis. The chlorophyll pigments in chloroplasts capture energy from photons, with this energy being used to synthesize organic molecules from carbon dioxide via the Calvin cycle. Large quantities of reactive oxygen species (ROS) and reactive nitrogen species (RNS) are generated during photosynthesis, especially under the stressful conditions such as exposure to excessive light intensity, heat, salt, cold, drought and environmental pollutants^1^,^2^,^3^,^4^. ROS and RNS, if not properly detoxified, damages chloroplasts and reduces their photosynthetic efficiency, ultimately killing the cell. This damage is referred to as oxidative stress.

During evolution, plants developed an array of mechanisms to protect themselves against oxidative stress^5^. One mechanism is the production of antioxidants; which include ascorbic acid, carotenoids, tocopherol, glutathione and polyphenols^6^. Interestingly another antioxidant, melatonin, initially thought to be exclusively an animal hormone, was identified in plants in 1995^7^,^8^. Since then melatonin has been detected in many different species of plants and plant products^9^,^10^,^11^,^12^,^13^. The significance of melatonin in plants is related to its potent free radical scavenging and antioxidant capacity^14^,^15^,^16^,^17^. In contrast to several other antioxidants, melatonin can enter every sub-cellular compartment due to its amphiphilic nature^16^. In addition, melatonin-related metabolites also function as antioxidants in what is referred to as the antioxidant cascade reaction. This cascade reaction allows melatonin and its derivatives to scavenge numerous radicals^16^. Melatonin application to leaves protects the chlorophyll from degradation induced by drought, delays senescence, and preserves the efficiency of photosystem II (Fv/Fm) when exposed to continuous dark stress^18^,^19^,^20^. During fruit development, endogenously-produced melatonin is negatively correlated with the amount of oxidized lipid^21^, suggesting that melatonin generated *de novo* can also reduce oxidative stress. In addition, we observed the sharp increase of melatonin level induced by high light intensity in summer day. Because the high light intensity resulted in ion leakage, therefore ROS over-production and oxidative damage in chloroplasts, it triggered our research on the melatonin synthesis in chloroplasts.

In comparison to animal cells, plant cells contain much higher levels of melatonin^16^. This raises the question as to whether animals and plants have different mechanisms for synthesizing this indole-containing compound. The penultimate enzyme in melatonin biosynthesis, serotonin *N*-acetyltransferase (SNAT), formerly arylalkylamine *N*-acetyltransferase (AANAT)^22^,^23^, may have different phylogenetic origins in animals and plants. Genetic analysis has shown that the DNA sequences of the plant *SNAT* gene is more closely related to that of cyanobacteria than to animal sequences^22^. Cyanobacteria have the capacity to synthesize melatonin and this trait may have passed to its descendent chloroplast. Taken together, it has been hypothesized that chloroplasts may be the primary site for melatonin biosynthesis^24^,^25^.

To test this hypothesis, *N-*acetylserotonin*-O-*methyltransferase *9,* from a salt tolerant apple species *Malus zumi (MzASMT9*), was cloned and confirmed to be localized to chloroplasts by *in vivo* fluorescence observations and Western blot. With the aid of molecular technologies combined with purification of chloroplasts, we confirmed that chloroplasts were able to synthesize melatonin.

## Results

### Melatonin biosynthesis occurs in purified chloroplasts

**Figure 1:**
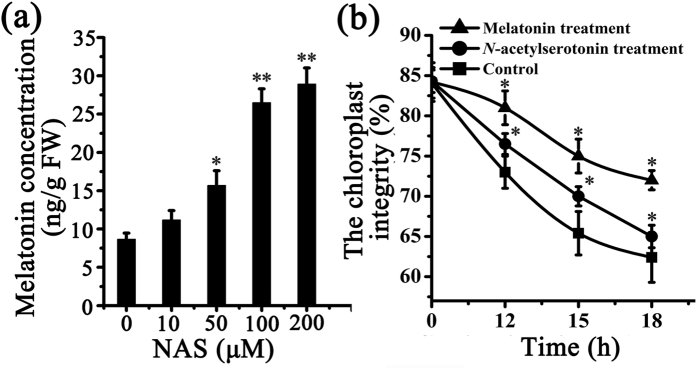
Melatonin production in purified *Arabidopsis* chloroplasts and the effect of melatonin on their integrity. (**a**) Different concentrations of *N*-acetylserotonin were added to purified *Arabidopsis* chloroplasts at 4 °C for 6 h. Melatonin concentration was quantified using HPLC. (**b**) The envelope integrity of chloroplasts with/without *N*-acetylserotonin (10 μmol/L) and melatonin (10 μmol/L) treatment at different time points. Asterisks (*) indicate significant differences from the control (*P < 0.05; **P < 0.01).

Chloroplasts were purified from *Arabidopsis* leaves and kept at 4 °C in darkness. The envelope integrity was tested^26^,^27^ and it was found that 83% of the purified chloroplasts were structurally intact. Melatonin was present in purified chloroplasts at a level of 0.1–10 ng/g FW. However, this does not exclude the possibility that the melatonin present has been transported into chloroplasts from other parts of the plant. To confirm that the melatonin is synthesized by chloroplasts *de novo*, different concentrations of *N*-acetylserotonin, the substrate of ASMT, were fed to purified chloroplasts. Melatonin production in *N*-acetylserotonin-fed chloroplasts was significantly higher (1.6 ± 0.05 ng/g FW) than that in control chloroplasts (0.8 ± 0.05 ng/g FW). Melatonin production exhibited an apparent dose-response change with the *N*-acetylserotonin concentrations (Fig. 1a). When the concentration of *N*-acetylserotonin was 200 μM, melatonin production (2.8 ± 0.05 ng/g FW) was about four times higher than that of control (0.8 ± 0.05 ng/g FW). The results show that chloroplasts have the capacity to synthesize melatonin, when provided with precursors.

### Protective effects of melatonin on purified chloroplasts

To elucidate the potential protective role of melatonin in chloroplasts, the envelope integrity of purified chloroplasts was recorded. The envelope integrity was 83% in freshly purified chloroplasts. During *in vitro* culture, the chloroplast envelope degraded progressively. For example, during storage of purified chloroplasts from 12 h to 18 h at 4 °C, the chloroplast integrity of control declined from 73% to 63%. The supplementation of 10 μM *N*-acetylserotonin (substrate of melatonin) in purified chloroplasts made the chloroplasts integrity slowly changed from 76.5% to 65% during storage. In contrast, in melatonin (10 μM) treated chloroplasts, the chloroplasts integrity declined even more slowly from 81% to 73% (Fig. 1b). This suggests that melatonin significantly delayed the degradation of purified chloroplasts.

### Phylogenetic tree construction and multiple alignments of MzASMT9

**Figure 2:**
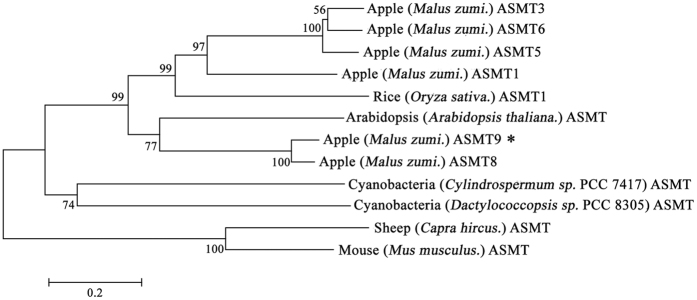
The phylogenetic tree was constructed using the neighbor-joining method and a bootstrap test with 1000 iterations, using MEGA5.2 software. The GenBank accession numbers are AK072740 (Rice, *Oryza sativa.* ASMT1), AT4G35160 (Arabidopsis, *Arabidopsis thaliana.* ASMT), KJ123721 (Apple, *Malus zumi*. ASMT1), KJ156528 (Apple, *Malus zumi*. ASMT3), KT633934 (Apple, *Malus zumi*. ASMT5), KJ156529 (Apple, *Malus zumi*. ASMT6), KJ156530 (Apple, *Malus zumi*. ASMT8), KJ156531 (Apple, *Malus zumi*. ASMT9), JF815374 (Goat, *Capra hircus.* ASMT), NP_001186141 (Mouse, *Mus musculus.* ASMT), AFZ23489 (Cyanobacteria, *Cylindrospermum stagnale.* PCC 7417 ASMT) and AFZ51979 (Cyanobacteria, *Dactylococcopsis salina.* PCC 8305 ASMT).

To identify candidate genes encoding for ASMT, the terminal enzyme which catalyzes melatonin synthesis from *N*-acetylserotonin, sequences with similarity to *ASMT* genes from *Oryza sativa* were used in a BLAST-based search of the apple genome. A total of six candidate genes were identified. The phylogenetic tree was constructed to identify their relationship with the *ASMT* gene in the chloroplast progenitor, cyanobacteria. The result showed that all the plant ASMTs including MzASMT9 have the closer relationship with that of the cyanobacteria, rather than to the lineage of animal ASMTs (Fig. 2). This suggests its origin has a link to cyanobacteria. The multiple alignment results indicate that MzASMT9 has the functional domain shared by the O-methyltransferases of cyanobacteria (Fig. S1).

### MzASMT9 was localized in chloroplasts

**Figure 3:**
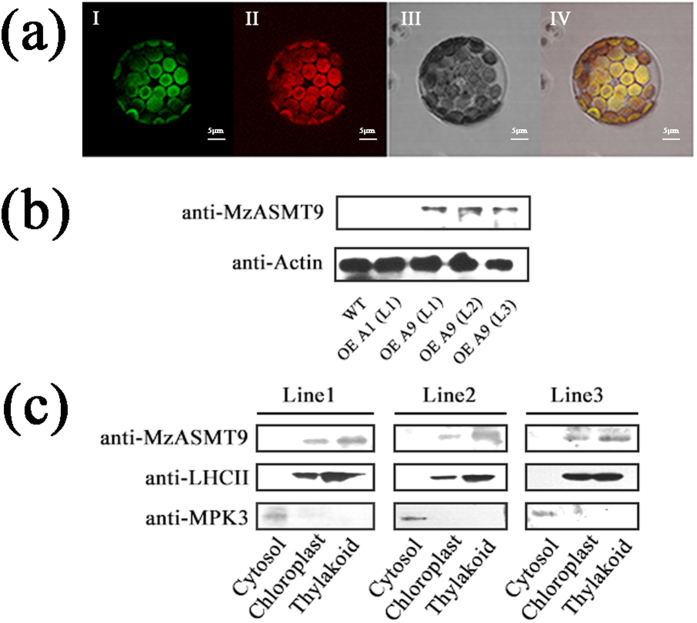
The localization of MzASMT9 protein in chloroplasts. (**a**) The localization of MzASMT9-GFP in protoplasts of transgenic *Arabidopsis* ectopically expressing *MzASMT9*. It was visualized by confocal microscopy (60 × 10). The green fluorescence from the MzASMT9-GFP was shown in picture I. The chloroplasts were marked by the red fluorescence in picture II. The protoplast was observed under bright field in picture III. The green fluorescence and red fluorescence merged together in picture IV under bright field. (**b**) The specificity of anti-MzASMT9, as determined using Western blot of total protein from wild type and transgenic lines ectopically expressing *MzASMT1* and *MzASMT9*. (**c**) The sub-organelle localization of MzASMT9-GFP in different transgenic *Arabidopsis* lines ectopically expressing *MzASMT9* as determined by Western blot with antibodies of anti-MzASMT9, anti-LHCII or anti-MPK3. The anti-MPK3 antibody was used as a cytosolic marker; anti-LHCII was used as a thylakoids marker.

The on-line analysis (http://www.cbs.dtu.dk/services/TargetP/) of MzASMT9 protein suggested that it potentially localizes to the chloroplasts. To confirm this, the *MzASMT9* gene fused to GFP and was transformed into *Arabidopsis*. After the T3 single-insertion homologous transgenic lines were achieved, the protoplasts were isolated from one-month-old leaves of transgenic *Arabidopsis.* MzASMT9-GFP was observed to be co-localized with the red auto-fluorescence of chlorophyll using confocal microscopy (60 × 10) (Fig. 3a). In addition the 35S:MzASMT9-GFP was also introduced into tobacco leaves by agrobacterium-mediated transient transformation. Three days later, MzASMT9-GFP was observed to be located in chloroplasts under the confocal microscopy (Fig. S2a). Western blot was applied to further confirm the confocal result. Firstly, the specificity of MzASMT9 antibody was checked in the wild type and transgenic *Arabidopsis* lines ectopically expressing different genes (MzASMT1 and MzASMT9) (Fig. 3b). Then the Western blot analysis confirmed that MzASMT9 protein was localized in the thylakoids of the chloroplasts in the three transgenic *Arabidopsis* lines ectopically expressing *MzASMT9* gene (Figs 3c and S2). The direct co-localization of MzASMT9 with chlorophyll combined with the result of Western blot suggested that the MzASMT9 protein is localized in chloroplasts.

### The expression of *MzASMT9* gene and melatonin biosynthesis is up-regulated by high light intensity

**Figure 4:**
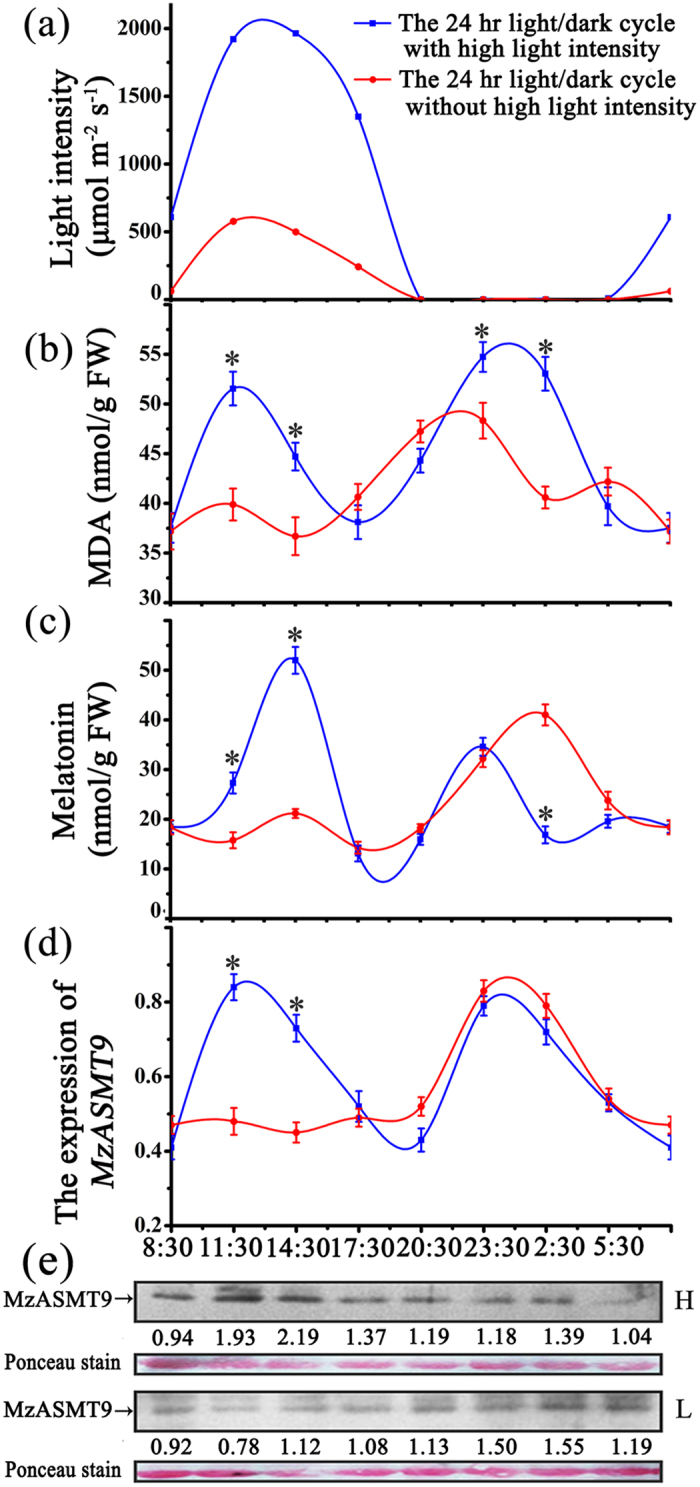
The effect of natural light on the expression of *MzASMT9* gene in apple leaves and its correlation with MDA and melatonin concentrations in 24 light/dark cycle. (**a**) The record of light intensities from 8:30, 17^th^ July 2015 to 5:30, 18^th^ July 2015 and 4^th^ August 2015 to 5^th^ August 2015 at the sampling times. (**b**) and (**c**) The levels of MDA and melatonin in 24 light/dark cycle. (**d**) and (**e**) The expression profiles of *MzASMT9* gene and MzASMT9 protein of apple leaves in 24 h light/dark cycle with high intensity of light (labeled with H) and low intensity of light (labeled with L). The arrows indicate the MzASMT9 protein.

To test the hypothesis that melatonin protects chloroplasts from oxidative stress when the plants are exposed to light, melatonin concentrations in leaves (*Malus zumi*) were measured under different light intensities. Concurrently, the expression of the MzASMT9 using qPCR and Western blot was monitored. When the plants were exposed to a weak intensity of light (498 μmol m^−2^ s^−1^ –577 μmol m^−2^ s^−1^), *MzASMT9* gene expression and melatonin production (about 20 ng/g FW) was significantly lower than that in leaves (about 50 ng/g FW) of plants which were exposed to high intensity of light (1920–1963 μmol m^−2^ s^−1^)^28^ (Figs 4 and S3). Also, the high intensity light exposure was associated with the increased malondialdehyde (MDA) levels indicating increased oxidative stress (Fig. 4b).

### The protein expression, purification and enzyme kinetics of MzASMT9

**Figure 5:**
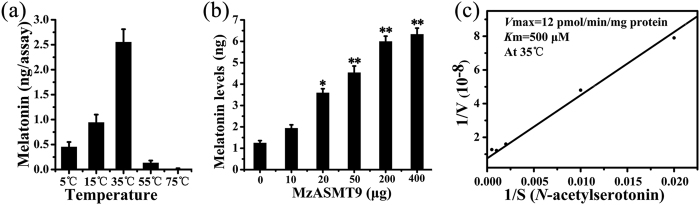
The kinetics of MzASMT9 protein. (**a**) The influence of temperature on the activity of purified recombinant MzASMT9 protein. (**b**) The effect of different doses of MzASMT9 protein on melatonin production. Data are means ± SD of triplicate experiments. (**c**) The standard curve of *K*_m_ and *V*_max_ of MzASMT9 protein. The MzASMT9 enzyme was incubated with various concentrations of *N*-acetylserotonin for 60 min at 35 °C. *K*_m_ and *V*_max_ were determined using Lineweaver–Burk plots.

*MzASMT9* was cloned from *Malus zumi* and expressed in *E. coli*. The MzASMT9 protein was purified using a GST tag. The enzyme activity of purified MzASMT9 was tested for its ability to convert its substrates, *N*-acetylserotonin and S-adenosyl-L-methionine, to melatonin. The temperature dependency of the enzyme activity of purified recombinant MzASMT9 protein was measured. The maximal activity of this enzyme was observed to be 35 °C (Fig. 5a). This is different from that of rice ASMT in which the optional temperature is around 55 °C^25^. However, at 55 °C, MzASMT9 still retains partial activity. The calculated *K*_m_ and *V*_max_ of the purified recombinant MzASMT9 at 35 °C were 500 μM and 12 pmol/min·mg protein, respectively (Fig. 5c).

When a temperature of 35 °C was selected (the most suitable temperature for the enzymatic reaction of MzASMT9 protein) with a reaction period of 1 h, it appeared that there was a concentration-response relationship between MzASMT9 protein and melatonin production (Fig. 5b). This dose-response relationship also applied to *N*-acetylserotonin (a substrate of ASMT) concentration (Fig. 5c). These results suggest the MzASMT9 protein can catalyze melatonin synthesis.

### Overexpression of *MzASMT9* in *Arabidopsis* increases melatonin concentrations, photosynthetic rate, fresh biomass and dry biomass

**Figure 6:**
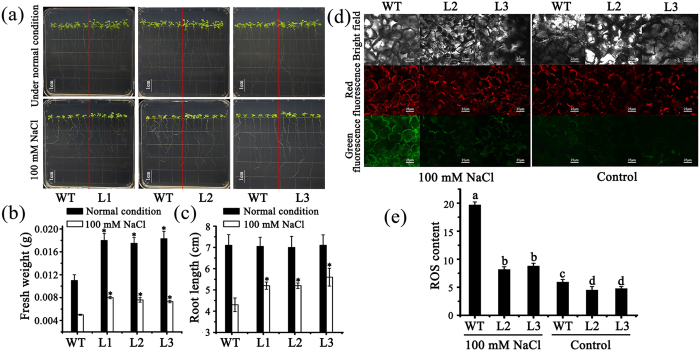
The MzASMT9-GFP conferred transgenic *Arabidopsis* enhanced salt tolerance via efficient ROS mitigation. (**a**) The phenotype of wild type and three transgenic *Arabidopsis* lines growing on MS media (first line) and MS media supplemented with 100 mM NaCl (second line). (**b**) and (**c**) The fresh weight and root length in wild type and transgenic plants on MS media and MS media supplemented with 100 mM NaCl. (**d**) and (**e**) The ROS level in the chloroplasts of wild type and transgenic plants on MS media and MS media supplemented with 100 mM NaCl. Under the confocal microscope (40 × 10), the green fluorescence shows the ROS level dyed by CM-H_2_DCFDA (488 nm) and the red fluorescence marks the chloroplast (543 nm). Asterisks (*) and different letters indicate significant differences from the control (*P < 0.05).

**Figure 7:**
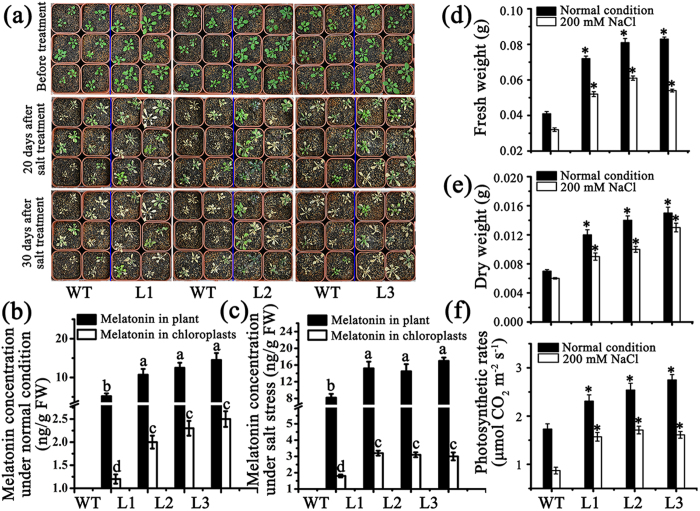
Melatonin concentrations in the chloroplasts and the entire plants, photosynthetic rates and fresh weights of wild type (WT) and three transgenic *Arabidopsis* lines (L1, L2 and L3) ectopically expressing *MzASMT9* under normal growth conditions and salt stress in soil. (**a**) The phenotype of wild type and transgenic plants under normal condition and under salt stress, 20 to 30 days after treatment. (**b**) and (**c**) The melatonin concentrations in the chloroplasts and the entire plants under normal condition or under salt stress for 20 days. (**d**,**e** and **f**) The fresh weight, dry weight and photosynthetic rate of wild type (WT) and three transgenic *Arabidopsis* lines (L1, L2 and L3) ectopically expressing *MzASMT9* under normal growth conditions and under salt stress in soil for 20 days Asterisks (*) and different letters indicate significant differences from the control (*P < 0.05).

To test the effects of the *MzASMT9* gene on melatonin synthesis and photosynthesis in chloroplasts, a total of three homozygous *Arabidopsis* lines with single insertion ectopically expressing *MzASMT9-GFP* gene were selected according to the Western blot results (Fig. 3b). The biomass of transgenic *Arabidopsis* was significantly increased compared with wild type on MS media/in soil (Figs 6a and 7a). In the MS media, the fresh biomass of the transgenic lines was significantly higher than the wild type, but no root length difference were observed (Fig. 6b,c). When the transgenic lines and wild type were grew in soil, the melatonin concentrations of the whole transgenic plants and their chloroplasts were significantly higher than that in wild type (Fig. 7b). The melatonin content of Line 3 was as high as 14.5 ng g^−1^ FW, approximately three times of control lines (5.2 ng g^−1^ FW). The *MzASMT9* expression and melatonin content also exhibited a good correlation with photosynthetic rate, fresh biomass and dry biomass. For example, the photosynthetic rate of Line 3 (2.75 μmol CO_2_·m^−2^·s^−1^) was higher than that of the wild type (1.73 μmol CO_2_·m^−2^·s^−1^). Accordingly, its fresh weight (0.08 g) and dry weight (0.015 g) were higher than that of the wild type (fresh weight 0.04 g, dry weight 0.007 g) (Fig. 7d,e,f).

### *MzASMT9* is induced by salt stress and confers enhanced salt tolerance by reducing excessive ROS

**Figure 8:**
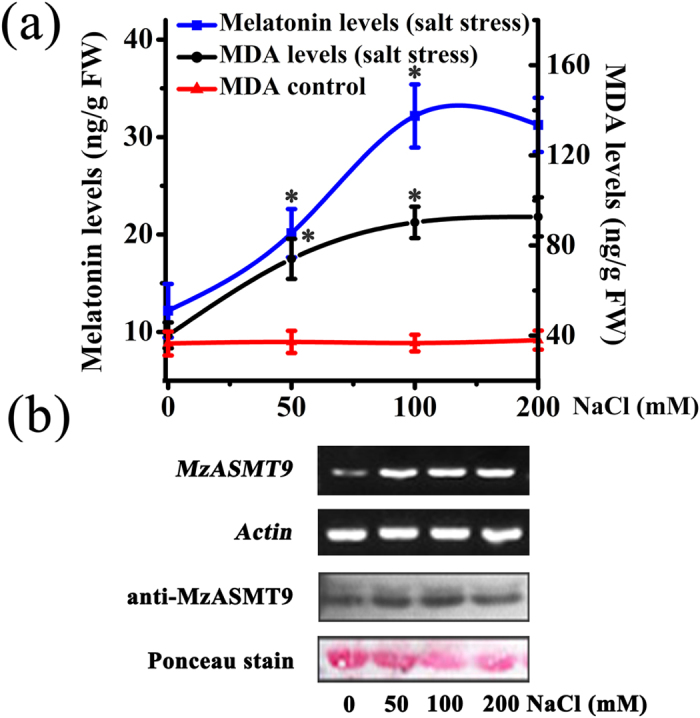
The melatonin concentrations, MDA contents, and *MzASMT9* expression in leaves of apple trees under salt stress. (**a**) The melatonin concentration and MDA levels in leaves of *Malus zumi*. under different concentrations of NaCl. (**b**) The cropped images indicated the expression of *MzASMT9* at mRNA level by semi-quantitative RT-PCR and MzASMT9 protein level detected by Western blot with the loading control dyed by ponceau.

RT-PCR and Western blot revealed that *MzASMT9* is induced by salt stress, and is accompanied by elevated MDA levels in apple leaves. The up-regulated *MzASMT9* also correlates with a marked rise in melatonin production in apple leaves (from 12 ng g^−1^ FW to 31 ng g^−1^ FW) (Figs 8 and S4). To identify the role of *MzASMT9* in melatonin production and its ability to detoxify excessively produced ROS under salt stress, the melatonin content, redox state and photosynthetic rate of chloroplasts, as well as the biomass and the phenotype of *Arabidopsis* transgenic plants was investigated. The transgenic *Arabidopsis* (Line 1–3) exhibited enhanced salt tolerance when grown in media. Under salt stress, the fresh weight and the root length of these transgenic plants were significantly higher than wild type, while the ROS levels in the chloroplasts of transgenic plants were significantly lower than the wild type plants (Fig. 6d,e). In the soil, the transgenic *Arabidopsis* (Line 1–3) also exhibited enhanced salt tolerance to salt treatment. After 20 days’ salt treatment in soil, the melatonin concentrations in the chloroplasts of transgenic *Arabidopsis* lines were 1.5–2.0 times of that in the wild type (Fig. 7c). The transgenic *Arabidopsis* exhibited a higher photosynthetic rate, had a higher biomass than those of the wild type. The photosynthetic rate of the Line three was approximately 1.61 μmol CO_2_·m^−2^·s^−1^ compared with 0.87 μmol CO_2_·m^−2^·s^−1^ for control lines. The fresh and dry weight of these transgenic plants was significantly higher than that of controls under salt stress, the fresh weight of Line 3 was nearly two times that of wild type (0.054 g compared with 0.032 g), the dry weight of Line 3 (0.013 g) was also around two times of wild type (0.006 g) (Fig. 7d,e).

## Discussion

Melatonin has been, until recently, considered as a hormone exclusive to animal systems^7^,^8^,^16^. Recent studies have focused on the biological effects of melatonin in plants. Melatonin promotes the development of lateral and adventitious roots, increasing plant seed germination rate, enhancing crop yield and delaying plant flowering^18^,^29^,^30^. In addition, as an antioxidant, melatonin has potent protective actions against a variety of abiotic and biotic stressors including heat, cold, drought, high salinity, soil pollutants and microorganism infections^19^,^31^,^32^,^33^,^34^,^35^.

Because of the sessile nature of plants, they are exposed to environmental stress of which animals can sometimes avoid. Thus, plants can suffer more stress-mediated oxidative damage caused by excessive production of ROS. Therefore, plants have multiple mechanisms to protect against stress including a higher melatonin production compared to that of animals. We explored the site of melatonin biosynthesis in plants; in particular, we targeted the chloroplasts. It is well known that chloroplasts were evolutionarily-derived from cyanobacteria during the process of endosymbiosis^24^. Melatonin and its synthetic enzymes have been identified in cyanobacteria^36^,^37^. Thus, it is reasonable to postulate that chloroplasts may have retained the melatonin synthetic trait that was present in cyanobacteria^24^. Recently, *N*-serotonin acetyltransferase (SNAT), the enzyme required for *N*-acetylserotonin synthesis, the direct precursor of melatonin, was reported to be localized in chloroplasts of rice plant^38^,^39^. We found that chloroplasts synthesize melatonin *in vitro*. To further show that chloroplasts are a site of melatonin synthesis it was necessary to prove that ASMT, the final enzyme in this pathway, was present in this organelle. For this purpose, an apple gene, MzASMT9 was cloned and *MzASMT9-GFP* was ectopically expressed in *Arabidopsis*. Using confocal microscopy, MzASMT9 was found to be localized in the *Arabidopsis* chloroplast. Western blot also confirm that MzASMT9 was localized in the *Arabidopsis* chloroplast thylakoid (Fig. 3). The level of MzASMT9 correlated with significantly higher melatonin production suggesting the expressed enzyme possess melatonin biosynthetic activity (Fig. 7b).

To study the kinetics of this enzyme, MzASMT9 protein was expressed and purified from *E. coli.* The results showed that the MzASMT9 enzyme can catalyze melatonin synthesis and had a dose-response relationship with its substrate *N*-acetylserotonin (Fig. 5). The *K*_m_ and *V*_max_ of the purified recombinant MzASMT9 were 500 μM and 12 pmol·min^−1^·mg^−1^ protein, respectively, at 35 °C. These data suggest that the MzASMT9 protein can catalyze melatonin synthesis.

The biological significance of melatonin synthesis in chloroplasts was examined. Photosynthesis requires light; however, excessive light intensity is a major factor in free radical generation. The chloroplastic synthesis of melatonin provides one possible protection for chloroplasts from oxidative stress. In the current study, all the transgenic *Arabidopsis* lines produced increased amounts of melatonin (Fig. 7). As a result, their chloroplasts exhibited an improved redox state with lower ROS levels under salt stress compared to the wild type (Fig. 6d,e). The high melatonin content and low levels of ROS resulted in the transgenic plants being more tolerant to salt stress. This was indicated by the enhanced photosynthetic efficiency and higher biomass of the transgenic plants compared to the wild type under salt stressed conditions (Fig. 7). In addition, the expression of *MzASMT9* was significantly induced by high light-intensity (around 2000 μmol m^−2^ s^−1^) (Fig. 4). This is different from animals where high light intensity universally inhibits nocturnal pineal melatonin production^16^. Plants exposed to light of high intensity, which induces oxidative damage due to the generation of singlet oxygen and H_2_O_2_^40^. Induction of melatonin by high light may mitigate the high oxidative damage caused by excessive ROS. This renders plants more resistant to high intensity light irradiation.

The regulatory mechanisms inducing *MzASMT9* gene expression are unknown; more studies are required to clarify the molecular signal transduction pathway. Importantly, melatonin synthesis in chloroplasts is stimulated by other stress. Salt stress significantly increased apple leaf lipid peroxidation while melatonin production was up-regulated to counteract the oxidative stress (Fig. 8a). It suggests that during the course of terrestrial plant evolution the melatonin biosynthetic pathway has become stress-inducible. The significance of melatonin production in chloroplasts appears to be to protect this organelle against oxidative stress including from excessive light irradiation, high salinity, high temperature^41^. These new findings have important applications in agriculture, e.g., for increasing crop yield. Moreover, melatonin enrichment may well allow economically important plants to be grown in areas where they are subjected to extreme abiotic and biotic stress.

## Materials and Methods

### Plant material and growth conditions

Seeds of *Malus zumi* were sown in wet vermiculite in an environmentally-controlled growth chamber. After three weeks, the seedlings with three-to-four leaves were watered with complete nutrient solution. The temperature was at a constant 22 ± 2 °C under a light intensity of approximate 100 μmol m^−2^ s^−1^.

Surface-sterilized *Arabidopsis* seeds of wild-type (Col-0), and transgenic *Arabidopsis* lines ectopically expressing *MzASMT1* and *MzASMT9* were sown on MS media at pH 5.8 with 30 g L ^−1^ sucrose and 6 g L ^−1^ agar (>1300 g/cm^2^) and subjected to a 3 days dark treatment at 4 °C to synchronize their germinations. The seedlings were grown on MS media for 10 days and then they were planted in soil. The temperature of growth chamber was controlled at 22 ± 2 °C with a 16/8 h light/dark cycle and the light intensity was approximately 100 μmol m^−2^ s^−1^.

### Isolation of the chloroplasts

The method for *Arabidopsis* chloroplasts isolation was modified from Triboush and Nishimura *et al*. (1998)^42^,^43^. A total of 20 g six-week-old *Arabidopsis* leaves were ground with 30 mL pre-cooled chloroplast extract buffer [0.33 M sorbitol, 0.05 M MES (pH 6.1), 0.01 M NaCl, 2 mM MgCl_2_·6H_2_O, 2 mM EDTA-Na_2_, 0.5 mM KH_2_PO_4_, 2 mM sodium ascorbate] filtered with 4 × gauze and centrifuged at 3,200 rpm (0 °C) for 5 min. The precipitate was re-suspended in 1 mL pre-cooled suspension buffer [0.33 M sorbitol, 0.05 M HEPES (pH 7.6), 0.01 M NaCl, 2 mM MgCl_2_·6H_2_O, 2 mM EDTA-Na_2_, 0.5 mM KH_2_PO_4_, 2 mM sodium ascorbate]. A total of 1 mL pellets were carefully transferred into a tube with 3 mL of 80% percoll at the bottom and 3 mL 40% percoll as the intermediate layer. Finally, these tubes were centrifuged at 3000 rpm (0 °C) for 3 min. The green intact chloroplasts in the intermediate layer were collected. The purified chloroplasts were cultivated in the suspension buffer at 4 °C in darkness.

### Determination of the envelope integrity of the purified chloroplasts

Envelope integrity rate (%) = (Hill reaction activity in broken chloroplast − Hill reaction activity in intact chloroplast)/Hill reaction activity in broken chloroplast × 100. The Hill reaction in broken or intact chloroplasts was tested as described by Krause and Heber *et al*. (1975)^26^,^27^. Each experiment was independently repeated three times.

### Melatonin extraction and detection in chloroplasts

The chloroplasts purified from 4 g leaves of six-week-old *Arabidopsis* were ground into a fine powder, which was suspended in 10 mL methanol and ultra-sonicated (80 Hz) for 35 min at 45 °C. Then the melatonin was extracted and detected by HPLC as described by Zhao *et al*.^21^ (Fig. S5). Each experiment was independently repeated three times.

### Functional assays in purified chloroplasts

The chloroplasts from six-week-old *Arabidopsis* leaves were divided into five groups. Group I was cultured at 4 °C for 6 h as control. Group II to Group V were co-cultured with different concentrations (10 μM, 50 μM, 100 μM and 200 μM) of substrate, *N*-acetylserotonin, at 4 °C for 6 h. Melatonin in chloroplasts was extracted and measured as mentioned previously. Each experiment was independently repeated three times.

The purified chloroplasts from six-week-old *Arabidopsis* leaves were divided into ten groups. Group I was the fresh purified chloroplasts. Group II to Group IV were the *in vitro* purified chloroplasts without exogenous organism. Group V to Group VII were the *in vitro* purified chloroplasts with 10 μmol/L exogenous *N*-acetylserotonin, Group VIII to Group X were the *in vitro* purified chloroplasts with 10 μmol/L exogenous melatonin. The envelope integrity of Group II, V and VIII was detected at 12 h after cultivation, Group III, VI and IX at 15 h, Group IV, VII and X at 18 h following the method described above. Each experiment was independently repeated three times.

### The phylogenic tree construction of ASMTs from different species and the multiple alignments of MzASMT9

The homologous genes of *ASMT* in apple were blasted in NCBI. In order to find the hierarchy of *MzASMT9* gene in evolution, a phylogenic tree was constructed using the amino acid sequences encoded by these homologous genes from cyanobacteria, rice, Arabidopsis, goat and mouse. The multiple alignments were conducted using DNAMAN to identify the shared domains among the proteins encoded by the homologous genes of cyanobacteria, rice, Arabidopsis, goat and mouse.

### Determination of subcellular localization of MzASMT9

The on-line analysis (http://www.cbs.dtu.dk/services/TargetP/) of six MzASMT proteins was applied to determine whether there is any ASMT localized in chloroplast. The coding sequence of *MzASMT9* without stop codon was sub-cloned in the Gateway® pGWB405 vector to generate a fusion protein with C-terminal GFP (Green Fluorescent Protein)-tag. The *MzASMT9* gene fused to GFP and was introduced into *Arabidopsis*. T3 single-insertion homologous transgenic lines were achieved. The protoplasts were isolated from one-month-old leaves of transgenic *Arabidopsis* as described by Li *et al*.^44^. The subcellular localization of MzASMT9-GFP was observed by Olympus confocal microscopy (60 × 10) (OLYMPUS, Tokyo, Japan). The images were processed with the FV10-ASM software version 3.0. The pGWB405-MzASMT9-GFP plasmids were introduced into the *Agrobacterium tumefaciens* strain GV3101. One-month-old *Nicotiana benthamiana* leaves were infiltrated with GV3101 carrying *35S:MzASMT9-GFP*. The subcellular localization of MzASMT9 was observed by Olymbus confocal microscope (40 × 10) (OLYMBUS, Tokyo, Japan) three days after transient transformation. The images were processed with the FV10-ASM software version 3.0.

### Determination of sub-localization of MzASMT9 in chloroplast

To prepare polyclonal antibody specific for MzASMT9 protein, a rabbit was immunized with purified MzASMT9 (2.4 mg/ml) emulsified with equal amount of Freud’s complete adjuvant via subcutaneous injection. The equal volume of antigen mixed with Freud’s incomplete adjuvant was injected every 10 days for four times. Antibody was isolated from neck artery blood of the immunized rabbit. All the methods were carried out in accordance with relevant guidelines and regulations and all the experimental protocols were approved by Beijing Municipal Science & Technology Commission. The chloroplasts and thylakoid were extracted from leaves of the one-month-old transgenic *Arabidopsis* lines ectopically expressing MzASMT9 by the modified method from Fristedt (2009)^45^. Western blot was applied with antibodies of anti-MzASMT9 (rabbit, 1:2000), anti-LHCII (rabbit, 1:3000) and anti-MPK3 (rabbit, 1:2000). Anti-MPK3 antibody was used as a cytosolic marker and anti-LHCII as a chloroplast marker. The secondary antibody used was Goat anti-rabbit IgG. The chemiluminescent signals were detected by a procedure using an ECL detection kit (Amersham-Pharmacia, USA). The loading control of the Western blot was stained by Coomassie Brilliant Blue.

### The specificity for the MzASMT9 antibody

To check the specificity of the antibody of MzASMT9, the total protein were extracted from the wild type and three transgenic *Arabidopsis* lines ectopically expressing *MzASMT9* and one transgenic line ectopically expressing *MzASMT1*^44^,^46^. Western blot was applied with antibodies of anti-MzASMT9 (rabbit, 1:2000) and antibody of anti-Actin (Mouse, 1:2000).

### Effect of light-intensity on MzASMT9 expression and melatonin synthesis

The leaves of apple trees were sampled at 8:30, 11:30, 14:30, 17:30, 20:30, 23:30 02:30, 05:30 respectively on a cloudy day on 17^th^ and 18^th^ (July) 2015 and the light intensities were 61 μmol m^−2^ s^−1^, 577 μmol m^−2^ s^−1^, 498 μmol m^−2^ s^−1^, 241 μmol m^−2^ s^−1^, 0 μmol m^−2^ s^−1^, 0 μmol m^−2^ s^−1^, 0 μmol m^−2^ s^−1^, 0 μmol m^−2^ s^−1^, respectively. Another set of samples were harvested at a sunny day (4^th^ and 5^th^ August 2015) with the light intensity of 608 μmol m^−2^ s^−1^, 1920 μmol m^−2^ s^−1^, 1963 μmol m^−2^ s^−1^, 1350 μmol m^−2^ s^−1^, 0 μmol m^−2^ s^−1^, 0 μmol m^−2^ s^−1^, 0 μmol m^−2^ s^−1^, 9 μmol m^−2^ s^−1^, respectively. The plants were located at latitude 40°00′N, longitude 116°35′W. A total of 15 g leaves were collected for each time point. The light intensity was measured at every sampling time point using a TES-1335 digital light meter (TES, Taipei, Taiwan).

Total RNA was isolated from leaves of apple trees, using EASY spin Plant RNA Rapid Extraction Kit (Biomed, Beijing, China) following the manufacturer’s protocol. The reverse transcription was performed following the protocol of the kit (Promega, Madison, USA). The *MzASMT9* gene specific primers (F: 5′-TGATCTGCCCCATGTCGT-3′ and R: 5′- CTTTGTGGCGAGGGAAAC-3′) were used for qRT-PCR. The *actin* gene was used as the internal standard. qRT-PCR was performed with SYBR Premix Ex Taq (Takara, DaLian, China) on an ABI 7500 real-time PCR machine (ABI, Carlsbad, USA).

The total protein was isolated from leaves of apple trees according to Wang *et al*.^47^. Western blot was applied with antibodies of anti-MzASMT9 (rabbit, 1:2000). The loading control was stained by ponceau.

The melatonin and malondialdehyde (MDA) detection in the leaf samples were conducted as described by Zhao *et al*.^21^. Each experiment was independently repeated at least three times.

### The cloning of *MzASMT9* gene and the expression of MzASMT9 protein in *E. coli*

The coding frame of *MzASMT9* was amplified and ligated into pMD 19-T Simple, which was then digested with BamHI/EcoRI and inserted into the pGEX-6p-1. The expression of MzASMT9 was induced by IPTG in *E. coli.* strain BL21 at 23 °C for 8 h incubation. The bacteria were centrifuged at 8,000 rpm for 10 min. The sediment was suspended and subjected to ultrasonication and then centrifuged at 10,000 rpm for 50 min at 4 °C. The GST-MzASMT9 protein was purified by precipitation with GSTrap^TM^ column (CWBIO, Beijing, China), which was equilibrated with a quintuple volume of PBS buffer. The purity of the GST-MzASMT9 protein was confirmed by SDS–PAGE.

### Determination of MzASMT9 enzyme activity

A total of 50 μg MzASMT9 protein purified from *E. coli.* was incubated with the substrates (0.5 mM S-adenosyl-L-methionine and 0.5 mM N-acetylserotonin) and 100 μL of 100 mM potassium phosphate buffer (pH 7.8). The enzyme reaction was carried out for 60 min at 30 °C and stopped by 50 μL methanol. A total of 200 μL acetonitrile was then added to precipitate the protein. The mixture was filtered with 0.1 μm filter. Finally, 800 μL of the filtered solution was injected into the HPLC for melatonin analysis. The enzyme activity was evaluated by the production of melatonin against the amount of purified MzASMT9 protein per minute. The experiment was independently repeated at least three times.

### The effects of different temperatures on MzASMT9 enzyme activity

To determine the effect of temperature on MzASMT9 enzyme activity, the activities of the purified recombinant MzASMT9 protein were assayed at various temperatures (5 °C, 15 °C, 35 °C, 55 °C and 75 °C, respectively). The enzymatic products were analyzed by HPLC as described above. The substrate affinity (*K*_m_) and maximum reaction rate (*V*_max_) values for MzASMT9 were determined at the optimum temperature (35 °C) and were calculated using Lineweaver–Burk plots. Each experiment was independently repeated three times.

### The dose-response effect of MzASMT9 protein on the melatonin production

The different amount of MzASMT9 protein (0 μg, 10 μg, 20 μg, 50 μg, 200 μg, 400 μg, respectively) was added to the reaction system. After 1 h reaction at 35 °C, the products were analyzed by HPLC as described above. Each experiment was repeated at least three times independently.

### The generation of *Arabidopsis* ectopically expressing *MzASMT9*

To confirm the function of *MzASMT9* in melatonin synthesis, *MzASMT9* gene was introduced into *Arabidopsis*. The *Arabidopsis* plants were transformed by the floral dip method using *Agrobacterium* GV3101 with 35S:MzASMT9-GFP. Western blot using MzASMT9 specific antibody mentioned above was applied to detect its expression in T3 single-insertion homologous transgenic lines.

### Measurements of biomass, root length and photosynthetic rate, melatonin in chloroplasts and entire plants, and redox states in chloroplasts of transgenic *Arabidopsis* under salt stress

A total of three single homozygous insertional lines with relatively high expression of *MzASMT9* were selected. After growing for 7 days at 22 °C with a 16/8 h light/dark cycle, half of the seedlings were transferred to MS media containing 100 mM NaCl in the square petri dish, while the other half of the seedlings were moved to fresh MS media. After 8 days, all the transgenic lines and wild type seedlings under normal growth condition and salt stress were harvested for fresh weight and root length measurement. They were also used for *in vivo* ROS detection in chloroplasts. The samples were incubated with 5-(and 6)-chlorom-ethyl-2′–7′-dichlorodihydrofluorescein diacetate acetyl ester (CM-H_2_DCFDA) for 20 min, then they were washed with distilled H_2_O to remove excess CM-H_2_DCFDA. The ROS levels of chloroplasts from salt-treated and control plants were measured as described by Guan *et al*.^48^. The green fluorescence shows the ROS level and the red fluorescence marks the chloroplast. The fluorescence images were observed using Olympus confocal microscope (40 × 10). The ROS content was measured by image J.

In addition to being cultured in media, 12-day-old plants of wild type and the plants from every transgenic line growing in MS media were transferred to soil. After 20 days, half of the plants were used for photosynthetic rate, fresh weight, dry weight measurement and melatonin detection in entire plants and purified chloroplasts. Then, the other half of the plants were treated with 200 mM NaCl. After 20 days’ salt treatment, these plants were also used for photosynthetic rate, fresh weight, dry weight measurement and melatonin detection in entire plants and in purified chloroplasts. The method for chloroplast isolation from *Arabidopsis* plants was described in Triboush and Nishimura *et al*.^42^,^43^ The photosynthetic rate was measured by LI-6400XT (LI-COR, Lincoln, USA) according to the producer’s protocol using the chamber for *Arabidopsis thaliana* leaves. The light intensity was at 300 μmol m^−2^ s^−1^. The humidity was about 50% and the temperature was 23 °C. The scanner was used to measure the leaf area. Each experiment was independently repeated at least three times.

### *MzASMT9* expression, melatonin and malondialdehyde (MDA) detection in apple leaves under salt stress

The leaves of one-month-old *Malus zumi.* plants grown in a seed chamber were treated with different concentrations of NaCl (0 mM, 50 mM, 100 mM and 200 mM). After the treatment of 12 h, a total of 2 g leaves were harvested for melatonin and malondialdehyde (MDA) detection as described by Zhao *et al*.^21^. A total of 1 g leaves was harvested for RNA extraction to analyze *MzASMT9* expression. The experiment was repeated at least three times. The RT-PCRs were performed with the *MzASMT9* and the *actin* gene specific primers were as described above. The PCR products were analyzed in 1% TAE-agarose gels stained by ethidium bromide. Western blot was applied with antibodies of anti-MzASMT9 (rabbit, 1:2000) as described above. The loading control was stained by ponceau. Each experiment was independently repeated three times.

### Statistical analysis

The data are expressed as means ± SD. One-way ANOVA was used for the normality evaluation followed by a Tukey-Kramer multiple comparison test P < 0.05 was considered as statistically significant. Statistical evaluations were carried using SPSS software (IBM, Armonk, NY, USA).

## Additional Information

**Accession codes:** The GenBank accession numbers are KJ123721 (Apple, Malus zumi. ASMT1), KJ156528 (Apple, Malus zumi. ASMT3), KT633934 (Apple, Malus zumi. ASMT5), KJ156529 (Apple, Malus zumi. ASMT6), KJ156530 (Apple, Malus zumi. ASMT8), KJ156531 (Apple, Malus zumi. ASMT9), AK072740 (Rice, Oryza sativa. ASMT1), AT4G35160 (Arabidopsis, Arabidopsis thaliana. ASMT), JF815374 (Goat, Capra hircus. ASMT), NP_001186141 (Mouse, Mus musculus. ASMT), AFZ23489 (Cyanobacteria, Cylindrospermum stagnale. PCC 7417 ASMT) and AFZ51979 (Cyanobacteria, Dactylococcopsis salina. PCC 8305 ASMT).

**How to cite this article**: Zheng, X. *et al*. Chloroplastic biosynthesis of melatonin and its involvement in protection of plants from salt stress. *Sci. Rep.* **7**, 41236; doi: 10.1038/srep41236 (2017).

**Publisher's note:** Springer Nature remains neutral with regard to jurisdictional claims in published maps and institutional affiliations.

## Supplementary Material

Supplementary Information
