# Supplementary material for: Chloroplastic biosynthesis of melatonin and its involvement in protection of plants from salt stress
Source: Sci Rep. 2017 Feb 1;7:41236. doi: 10.1038/srep41236 (PMC5286529; doi:10.1038/srep41236)
Supplement: Supplementary Information [file srep41236-s1.pdf]

Title: Chloroplastic biosynthesis of melatonin and its involvement in protection of plants from salt stress

Authors: Xiaodong Zheng[1,*], Dun X. Tan[2], Andrew C. Allan[3], Bixiao Zuo[1], Yu Zhao[1], Russel J. Reiter[2], Lin Wang[1], Zhi Wang[4], Yan Guo[4], Jingzhe Zhou[1], Dongqian Shan[1], Qingtian Li[1], Zhenhai Han[1] and Jin Kong[1,*]

Author affiliation:

[1]College of Agriculture and Biotechnology, China Agricultural University, Beijing, China

[2]Department of Cellular & Structural Biology, The UT Health Science Center, San Antonio, TX, USA

[3]Plant & Food Research, Private Bag 92169, Auckland, New Zealand;

[4]College of Biological Sciences, China Agricultural University, Beijing, China

Corresponding author:

Name: Jin Kong;

Address: No.2 Yuan Ming Yuan Xi Road, College of Agriculture and Biotechnology, China Agricultural University, Beijing, China

Email: jinkong@cau.edu.cn; *Tel/Fax:* +86-10-62734086; *Mobile:* +86-13331096169

Supplemental data

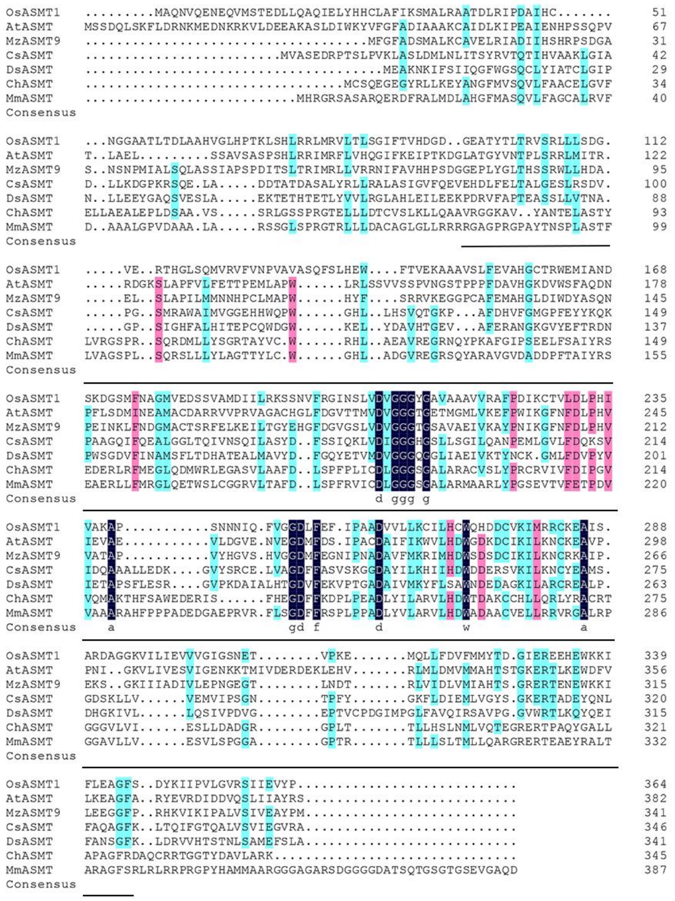

Supplemental Figure S1. The multiple alignments were constructed using DNAMAN (http://www.lynnon.com/). The shared domain by O-methyltransferases is underlined. The black color highlights the same sequence, while the pink color highlights the sequence with only one different amino acid and blue with more than one amino acid.

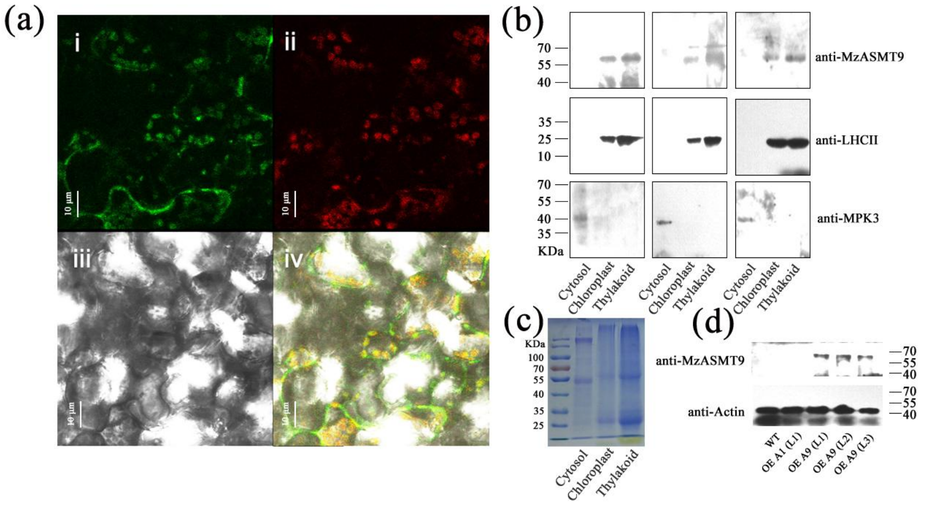

Supplemental Figure S2. (a) The localization of MzASMT9-GFP in the chloroplasts. (i) green fluorescence of MzASMT9-GFP; (ii) red fluorescence of chlorophyll; (iii) the bright-field image of *Agrobacterium tumefaciens*-infiltrated tobacco leaves; (iv) the two fluorescence images merged in bright-field. Tobacco leaves were infiltrated with *Agrobacterium tumefaciens* carrying MzASMT9-GFP driven by 35S promoter. After 3 days, the transgenic leaves were observed by confocal microscopy (40X10). It showed that the green MzASMT9-GFP protein co-localized with the chloroplasts marked by red. (b) The uncropped image of Western blot to confirm the localization of MzASMT9 protein in chloroplasts in three transgenic *Arabidopsis* lines ectopically expressing *MzASMT9*. (c) The loading control of the Western blot to indentify the sub-organelle localization of MzASMT9 protein was stained by Coomassie Brilliant Blue. (d) The uncropped image used to confirm the specificity of anti-MzASMT9, as determined using Western blot of total protein from wild type and transgenic lines ectopically expressing *MzASMT1* and *MzASMT9*.

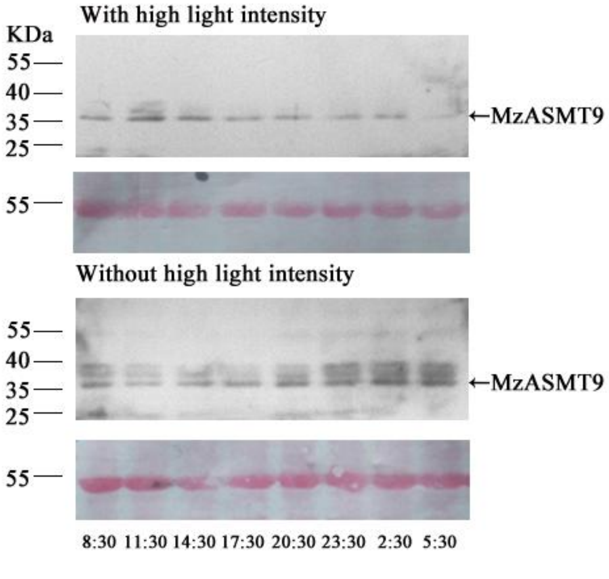

Supplemental Figure S3. The uncropped image of the MzASMT9 protein in 24 hr dark/light cycle with/without light of high intensity, the arrows indicate the position of MzASMT9. The loading control is stained by ponceau.

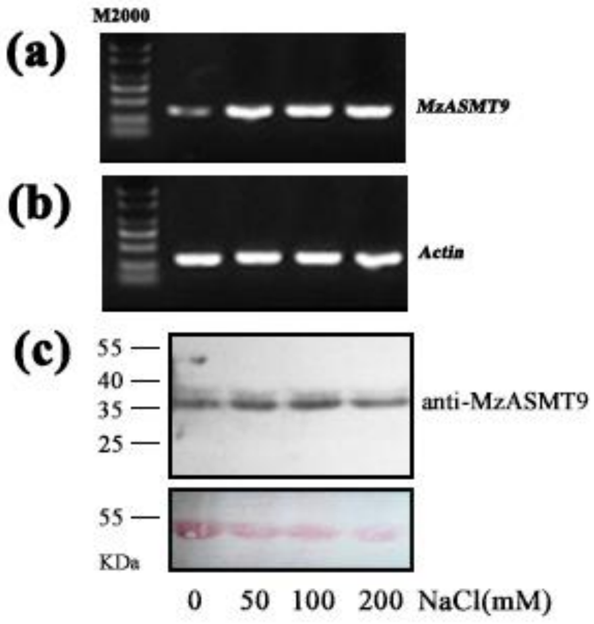

Supplemental Figure S4. The uncropped image for the expression detection of MzASMT9 at mRNA level and protein level under different treatment of salt stress.

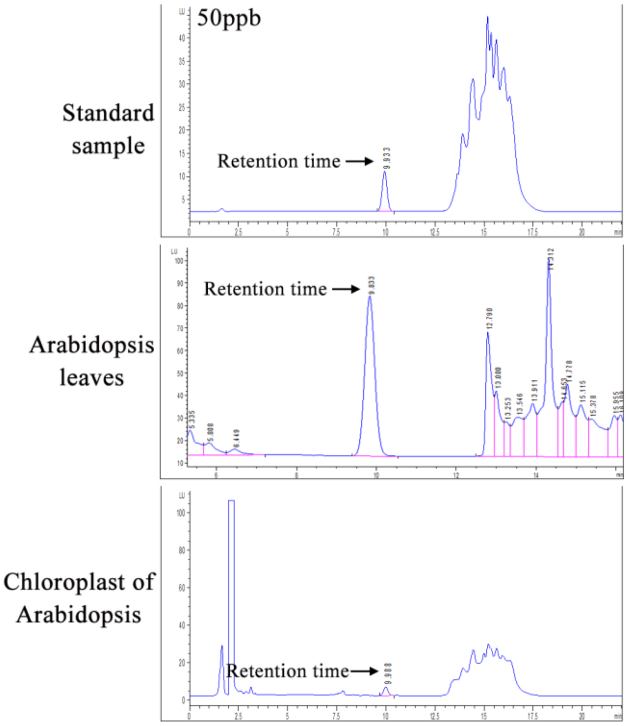

Supplemental Figure S5. HPLC spectrum of melatonin standard sample and the melatonin extracted from *Arabidopsis* leaves and chloroplasts. The arrows indicate the retention time at about 9.9 min.
